# MiR-139 suppresses β-casein synthesis and proliferation in bovine mammary epithelial cells by targeting the GHR and IGF1R signaling pathways

**DOI:** 10.1186/s12917-017-1267-1

**Published:** 2017-11-25

**Authors:** Yingjun Cui, Xia Sun, Lianfeng Jin, Guangpu Yu, Qingzhang Li, Xuejun Gao, Jinxia Ao, Chunmei Wang

**Affiliations:** 1 Key Laboratory of Dairy Science of Education Ministry, Northeast Agricultural University, Harbin, Heilongjiang 150030 People’s Republic of China; 2 College of Life Science, Northeast Agricultural University, Harbin, Heilongjiang 150030 People’s Republic of China

**Keywords:** miR-139, Mammary gland, β-casein, Growth hormone receptor, Type I insulin-like growth factor receptor

## Abstract

**Background:**

MicroRNAs have important roles in many biological processes. However, the role of miR-139 in healthy mammary gland remains unclear. The objective of this study was to investigate the effects of miR-139 on lactation in dairy cows.

**Results:**

Here, we found that miR-139 was down-regulated in mid-lactation dairy cow mammary tissues compared with mid-pregnancy tissues. Then, we prioritized two of potential target genes of miR-139 in cow, growth hormone receptor (*GHR*) and type I insulin-like growth factor receptor (*IGF1R*) for further functional studies based on their roles in lactation processes. Dual luciferase reporter assays validated direct binding of miR-139 to the 3**′**- untranslated region (UTR) of *GHR* and *IGF1R*. Moreover, over-expression or silencing of miR-139 affected mRNA levels of GHR and IGF1R in cultured bovine mammary epithelial cells (BMECs). Furthermore, over-expression of miR-139 decreased protein levels of β-casein, proliferation in mammary epithelial cell, and the protein levels of IGF1R and key members of the GHR or IGF1R pathways as well, whereas silencing miR-139 produced the opposite result. Among these signal molecules, signal transducer and activator of transcription-5 (STAT5), protein kinase B (also known as AKT1), mammalian target of rapamycin (mTOR), and p70-S6 Kinase (p70S6K) are involed in β-casein synthesis, and Cyclin D1 is involved in cell proliferation. In addition, silencing *GHR* decreased protein levels of β-casein, IGF1R, and key members of the IGF1R pathway, whereas co-silencing miR-139 and *GHR* rescued the expression of *GHR* and reversed *GHR* silencing effects.

**Conclusions:**

Our results demonstrate that *GHR* and *IGF1R* are target genes of miR-139 in dairy cow. MiR-139 suppresses β-casein synthesis and proliferation in BMECs by targeting the GHR and IGF1R signaling pathways.

## Background

MicroRNA-139-5p (miR-139-5p) and miR-139-3p are mature miRNAs generated from a miR-139 precursor [1, 2]. The knowledge about miR-139-3p function in bovine mammary epithelium is very limited, but it is worth noting that miR-139-3p has been identified in bovine milk [3]. It is a widely used assumption that there is only one type of mature bta-miR-139, and it has the same sequence as hsa-miR-139-5p. MicroRNAs regulate a wide variety of biological processes, including cell proliferation, apoptosis, developmental timing, and signal transduction [4]. Previous studies about miR-139 have focused on its antioncogenic and antimetastatic activities [5]. It has been shown that miR-139-5p suppresses proliferation, migration, and invasion in esophageal cancer [6], colorectal cancer [7], non-small cell lung cancer [8], hepatocellular carcinoma [9] and breast cancer [5, 10]. Moreover, miR-139-5p inhibits the epithelial-mesenchymal transition of hepatocellular carcinoma cells [2]. Although some microarray studies show that miR-139 expression is down-regulated in the mammary tissue during the transition period between pregnancy and lactation [11], little is known about effects of miR-139 in healthy mammary glands.

Growth hormone (GH) is a pituitary hormone that exerts a galactopoietic effect on the bovine mammary gland and controls milk protein synthesis [12, 13]. In addition to the pituitary gland, GH is produced in an autocrine manner by the mammary gland [14]. The effects of GH are mediated through interaction with growth hormone receptor (GHR), whose expression is regulated by GH [14, 15]. Localization of GHR is strong on the membrane as well as in the cytoplasm of the bovine mammary epithelial cell (BMEC) [15, 16]. GHR immunostaining within the nucleus is rare or absent [17]. GHR may be involved in milk protein secretion in BMECs [12]. The most abundant milk protein is β-casein, which makes up approximately 30% of the total protein in cows’ milk [18].

Type I insulin-like growth factor (IGF1) is the major mediator of GH effects in many tissues. Previous studies have indicated that GH can act indirectly on the mammary gland by stimulating IGF1 secretion from the liver [19]. Evidence has also shown that GH induces the expression of IGF1 mRNA in mammary gland [20, 21]. The functions of IGF1 are mainly mediated through the type I insulin-like growth factor receptor (IGF1R) [22]. Interestingly, some of the IGF1R downstream signaling members are shared with GH [20]. IGF1R signaling can promote cell proliferation, survival, differentiation, and protein synthesis [19, 20].

In this study, *GHR* and *IGF1R* were prioritized as two of potential target genes of miR-139 in cow. Combining their roles in lactation processes, and the down-regulation of miR-139 in the mammary tissue during the transition period between pregnancy and lactation, we hypothesized that miR-139 is also involved in regulation of lactation. The aim of this study was to investigate the effects of miR-139 on lactation in dairy cows.

## Methods

### Animals and mammary gland samples

Samples were collected from mammary glands of Holstein cows. All animals were clinically healthy. The average weights of the dairy cows were 609 ± 9.08 kg (mean ± SEM). The cows were separated into two groups by developmental stage: mid-pregnancy (*n* = 3) or mid-lactation (n = 3). Three pregnant cows went through a dry period and then were pregnant again. Lactating cows were at 90 DIM. The cows were slaughtered by exsanguination, and the mammary glands were removed immediately after slaughtering. Mammary gland samples were frozen in liquid nitrogen and stored at −80 °C. In our study, all animal experimental protocols were approved by the Institutional Animal Care and Use Committee of Northeast Agricultural University (China).

### Plasmid construction

The wild-type and mutated sequences of the 3**′**- untranslated region (UTR) of *GHR* (Cow *GHR*, NCBI Accession # NC_007318.5) and *IGF1R* (Cow *IGF1R*, NCBI Accession # AC_000178.1) with specific restriction sites *XhoI* and *NotI* were constructed by Sangon Biotech Company (Shanghai, China).

The 214-bp fragment from *GHR* 3′-UTR contained a predicted bta-miR-139 binding region (**5**′**-ACACG**GTGTAC**TGTAG-3**′). The binding site of the mutated 3′-UTR sequence of *GHR* was **5**′**-TGTGC**GTGTAC**ACATC-3**′**.** The 242-bp fragment from *IGF1R* 3′-UTR contained a predicted bta-miR-139 binding region (**5**′**-ACTGTAGA-3**′)**.** The binding site of the mutated 3′-UTR sequence of *IGF1R* was **5**′**-TGACATCT-3**′. All sequences were confirmed by sequence analysis. The wild-type or mutant 3′-UTR was subcloned into the *XhoI*-*NotI* site of the psiCHECK-2 vector (Promega, Madison, WI, USA).

### HeLa cell culture and dual luciferase reporter assay

The HeLa cell line was purchased from the American Type Culture Collection (ATCC, Rockville, MD, USA). The cell line was maintained in Dulbecco’s modified Eagle’s medium (DMEM) (High Glucose) (Invitrogen, Carlsbad, CA, USA) and supplemented with 10% fetal bovine serum (FBS) (Gibco, Rockville, Maryland, USA), penicillin (100 units/mL), and streptomycin (100 μg/mL) at 37 °C in a 5% CO_2_ humidified incubator. Cells were seeded at 1 × 10^5^ cells per well in a 12-well plate the day before transfection. Cells were co-transfected with a total of 500 ng of the psiCHECK-2 luciferase reporter construct or empty psiCHECK-2 vector, and 40 pmol of negative control of bta-miR-139 mimic or bta-miR-139 mimic (RiboBio Company, Guangzhou, China). Transfections were performed using Lipofectamine 2000. At 24 h post-transfection, the medium was changed. The cells were grown for an additional 24 h before the assay was performed.

Firefly and Renilla luminescent signals arising from psiCHECK-2 transfected cells were quantified according to the manufacturer’s instructions for the Dual-Luciferase Reporter Assay System (Promega, Madison, WI, USA) with a VICTOR Multilabel Counter luminometer (PerkinElmer, Waltham, MA). Independent experiments were performed 3–5 times.

### BMECs culture and transfection

BMECs were prepared and identified as previously described [23]. In brief, the tissue samples from lactating Holstein cows were minced with surgical scissors and transferred to cell culture bottles coated with collagen. Mammary tissue pieces were cultured in Dulbecco’s modified Eagle’s medium F-12 (DMEM/F12) (Gibco, Rockville, Maryland, USA) and supplemented with 10% FBS, penicillin (100 units/mL), and streptomycin (100 μg/mL) at 37 °C in a 5% CO_2_ humidified incubator. BMECs and fibroblasts were separated by selective trypsinization (0.25% trypsin). Pure BMECs were obtained after four passages and identified by immunofluorescence for Cytokeratin-18 and β-casein.

The BMECs were seeded at 3 × 10^5^ cells per well in a 6-well plate and grown for 24 h. Then, the cells were transfected with 100 pmol of negative control of bta-miR-139 mimic (Mimic NC), bta-miR-139 mimic, negative control of bta-miR-139 inhibitor (Inhibitor NC) or bta-miR-139 inhibitor (RiboBio Company, Guangzhou, China) using Lipofectamine 2000 (Invitrogen Life Technologies, Carlsbad, CA, USA) according to the manufacturer’s instructions. The control group was only transfected with Lipofectamine 2000. Opti-MEM (Reduced Serum Medium) (Gibco, Rockville, Maryland, USA) was used as medium. Approximately 5 h after transfection, the medium was changed to DMEM/F12 supplemented with 10% FBS.

### RNA extraction and quantitative real-time polymerase chain reaction assay

Total RNA was extracted from frozen tissues or BMECs using Trizol reagent (Invitrogen, Carlsbad, CA, USA) according to the manufacturer’s instructions. RNA extraction from BMECs was performed 24 h post-transfection. RNA integrity was assessed by electrophoresis on 1% (*w*/*v*) agarose gels. The ratio of the optical densities measured at 260 and 280 nm by an ND-1000 spectrophotometer (NanoDrop Technologies, USA) was >1.8 for all RNA samples.

To determine the relative amount of miR-139 or U6 transcripts, cDNA was synthesized using M-MLV reverse transcriptase (Invitrogen, Carlsbad, CA, USA) according to the manufacturer’s instructions. Primer sets used in reverse transcription and quantitative Real-time Polymerase Chain Reaction(qPCR) for bta-miR-139 or U6 were purchased from GenePharma Company (Shanghai, China). To determine the relative amount of *GHR* or *IGF1R* transcripts, cDNA was synthesized using the PrimeScript™ RT reagent kit (TaKaRa, Ostu, Japan). QPCR reactions were performed using SYBR Premix Ex Taq™ (TaKaRa, Ostu, Japan) and the ABI PRISM 7300 Real-Time PCR System (Applied Biosystems, Foster City, CA, USA). The primers were designed by Primer Premier 5.0 software. The primers used were as follows—*GHR*: forward 5′-CGTGGACAACGCTTACT -3′ and reverse 5′-AAGGGTTTCTGTGGTGAT -3′; *IGF-IR*: forward 5′-AGAAGCAGGCGGAGAAGGA-3′ and reverse 5′-AAGGGTTTCTGTGGTGAT -3′; *β-actin*: forward 5′-TTAGCTGCGTTACACCCTT -3′ and reverse 5′-GTCACCTTCACCGTTCCA -3′. All reactions were performed in triplicate. The expression of miR-139 was normalized to U6. Gene expression was normalized to β-actin. The stability of U6 and β-actin were confirmed under the experimental conditions. The relative levels of miRNA or mRNA were determined using the 2^-△△Ct^ method.

### Western blotting analysis

Western blotting analysis was performed as previously described [24]. Briefly, BMECs were harvested on ice using RIPA buffer (Beyotime Biotechnology, Shanghai, China) to which protease inhibitors had been added (2 mM PMSF, 5 mM Na_3_PO_4_, 5 mM NaF, and complete protease inhibitor) at 48 h post-transfection and then homogenized by ultrasonication. The BCA protein assay was used to measure protein concentration in the samples. Approximately 50 μg total protein was subjected to a 10% SDS-PAGE. The separated proteins were transferred to a nitrocellulose membrane and incubated in blocking buffer. Membranes were then incubated with primary antibodies overnight at 4 °C, followed by incubation with horseradish peroxidase-conjugated secondary antibodys against mouse, rabbit or goat (ZSGB-BIO, Beijing, China) at room temperature for 1 h. Protein bands were visualized using an ECL system. The following primary antibodies were purchased from Santa Cruz Biotechnology: antibodies for phosphorylated signal transducer and activator of transcription-5 (p-STAT5) (1:200 dilution, sc-11,761), signal transducer and activator of transcription-5 (STAT5) (1:200 dilution, SC-836), protein kinase B (also known as AKT1) (1:200 dilution, SC-1618), phosphorylated AKT1 (1:200 dilution, sc-135,650), phosphorylated p70-S6 Kinase (1:200 dilution, p-p706SK) (sc-11,759), p70-S6 Kinase (p706SK) (1:200 dilution, sc-230), Cyclin D1 (1:200 dilution, sc-718), and β-actin (1:400 dilution, sc-47,778); from Cell Signaling Technology: antibodies for IGF1R (1:1000 dilution, #3027), phosphorylated mammalian target of rapamycin (p-mTOR) (1:1000 dilution, #2971) and mammalian target of rapamycin (mTOR) (1:1000 dilution, #2972); and from Abbiotec Company: β-casein antibody (1:200 dilution, #251309). β-actin was used as an endogenous reference gene. All experiments were performed in triplicate. Western blotting data were scanned and quantified using Bandscan5.0 software.

### 3-(4,5-Dimethylthiazol-2-yl)-2,5- diphenyltetrazolium bromide assay

BMECs were cultured in 96-well plates (1 × 10^4^ cells per well). At 24, 48, 72, or 96 h post-transfection with Lipofectamine 2000 (Control), negative control of bta-miR-139 mimic (Mimic NC), bta-miR-139 mimic, negative control of bta-miR-139 inhibitor (Inhibitor NC) or bta-miR-139 inhibitor, cell proliferation was assayed using (3-(4,5-Dimethylthiazol-2-yl)-2,5- diphenyltetrazolium bromide) (MTT) dye (Amresco, Colorado, USA). MTT (5 mg/ml) was added to plates and incubated for 4 h. After that, the medium was discarded and cells were lysed in dimethyl sulfoxide (DMSO) (Amresco, Colorado, USA). Absorbance was measured at 490 nm using the Bio-Rad iMark 680 microplate reader (Bio-Rad, USA).

### Co-transfection of *GHR* siRNA and miR-139 inhibitor

Small interfering RNA (siRNA) duplexe targeting bovine *GHR* mRNA and a negative control were designed and synthesized by RiboBio Company (Guangzhou, China). The siRNA sequences were as follows: forward 5′-CCAGUCCUAGAGACAAAUU dTdT −3′ and reverse −3′ dTdT GGUCAGGAUCUCUGUUUAA 5′. The targeting sequence in GHR was as follow: CCAGTCCTAGAGACAAATT. BMECs were seeded in a 6-well plate at a density of 3 × 10^5^ cells per well in DMEM/F12 without antibiotics at ~80–90% confluence. They were co-transfected with negative control of bta-miR-139 inhibitor or bta-miR-139 inhibitor, and negative control of *GHR* siRNA or *GHR* siRNA (2 μg per 10^5^ cells). Lipofectamine 2000 was used according to the manufacturer’s protocol. Approximately 6 h after transfection, the medium was changed to DMEM/F12. RNA extraction from BMECs was performed 24 h post-transfection, and protein extraction was performed 48 h post-transfection.

### Statistical analysis

Statistical analyses were performed using SPSS19.0 software. All data are reported as the mean ± SEM of three independent experiments. Comparisons between treatments were made by *t* tests for two groups or ANOVA if there were more than two groups. Statistical significance was considered at *P*<0.05.

## Results

### Expression of miR-139 in dairy cow mammary tissues

**Fig. 1:**
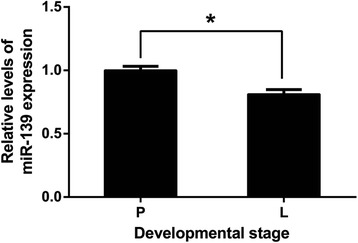
Expression of miR-139 in Holstein cow mammary gland. Mammary tissues were from mid-pregnancy (P) (*n* = 3) or mid-lactation (L) (n = 3) cows. MiR-139 expression was normalized to U6. Results are shown as means ± SEM from three independent experiments. Statistical analysis was conducted using the *t* test.“*”, statistical significance was considered at *P*<0.05

To determine the expression of miR-139 in dairy cow mammary glands, we used qPCR to measure levels of the mature miR-139 in mid-pregnancy and mid-lactation mammary tissues of dairy cows. U6 was used for normalization. The result showed that miR-139 expression in mid-lactation bovine mammary tissues was significantly reduced compared with that in mid-pregnancy tissues (*P* < 0.05) (Fig. 1). This result suggested that down-regulation of miR-139 may be important in lactation of dairy cows.

### MiR-139 directly targets 3′-UTRs of *GHR* and *IGF1R* in dairy cow

**Fig. 2:**
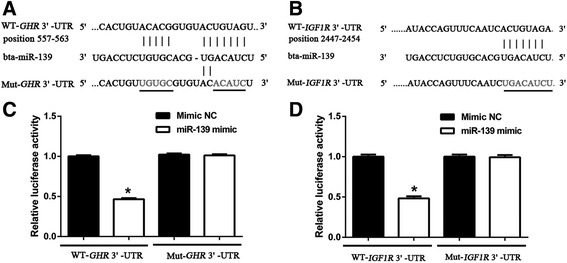
Identifying target genes of miR-139 via the dual luciferase reporter assay. **a** Bta-miR-139 binding sites in the 3**′**-UTR of *GHR*. Ten nucleotides (underlined) were mutated in luciferase reporter plasmids carrying *GHR* 3**′**-UTR. **b** Bta-miR-139 binding sites in the 3**′**-UTR of *IGF1R*. Eight nucleotides (underlined) were mutated in the luciferase reporter plasmids carrying *IGF1R* 3**′**-UTR. **c** Luciferase activity of reporter plasmids carrying wild-type or mutant *GHR* 3**′**-UTR in HeLa cells, in response to co-transfection with miR-139. **d** Luciferase activity of reporter plasmids carrying wild-type or mutant *IGF1R* 3**′**-UTR in HeLa cells, in response to co-transfection with miR-139. Results are shown as means ± SEM from three independent experiments. Statistical analysis was conducted using one-way ANOVA.“*”, statistical significance was considered at *P* < 0.05

Through a target prediction algorithm (TargetScan, http://www.targetscan.org), we found that *GHR* and *IGF1R* are potential target genes of miR-139 in cow. It is known that miRNAs function by binding to the 3**′**-UTR of their target mRNAs and inhibiting their expression. Then, to verify the binding of miR-139 to *GHR* and *IGF1R*, we performed a dual luciferase reporter assay in HeLa cells using the psiCHECK-2 vector and engineered luciferase reporter constructs containing the wild-type (WT) or mutant (Mut) 3′-UTR of the GHR or IGF1R gene, separately (Fig. 2a and b). Compared with co-transfection of negative control of miR-139 mimic with the luciferase reporter gene linked to the wild-type 3**′**-UTR of *GHR* or *IGF1R*, co-transfection of miR-139 mimic with the wild-type construct of *GHR* or *IGF1R* 3**′**-UTR strongly inhibited the luciferase activity. Luciferase activity was decreased in these cells by 53.5% or 51.7%, respectively (*P* < 0.05) (Fig. 2c and d). In addition, luciferase activity was not altered by co-transfection of negative control of miR-139 mimic/miR-139 mimic with the mutant construct of *GHR* or *IGF1R* 3**′**-UTR in which the miR-139 binding sequence was mutated (*P* > 0.05) (Fig. 2c and d). These results revealed that miR-139 directly binds to the 3**′**-UTRs of *GHR* and *IGF1R*.

### MiR-139 decreases mRNA levels of *GHR* and *IGF1R* in BMECs

**Fig. 3:**
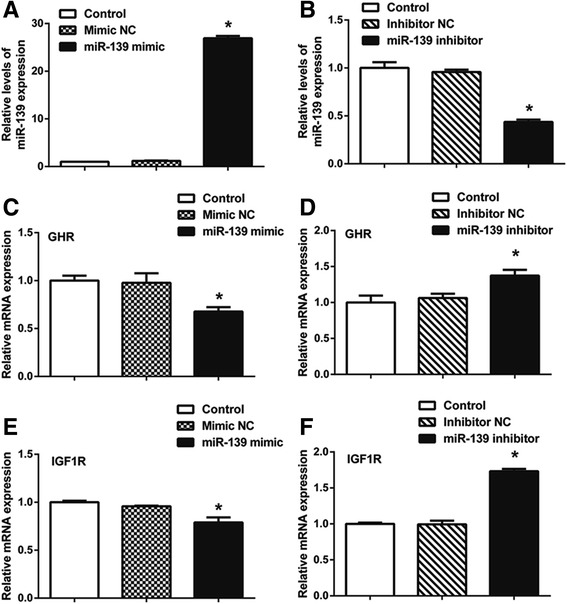
*GHR* and *IGF1R* are target genes of miR-139. BMECs were transfected with negative control of miR-139 mimic (Mimic NC), miR-139 mimic, negative control of miR-139 inhibitor (Inhibitor NC) or miR-139 inhibitor using Lipofectamine 2000. The Control group was only transfected with Lipofectamine 2000. MRNA levels of miR-139 (**a**, **b**), *GHR* (**c**, **d**) and *IGF1R* (**e**, **f**) were examined by qPCR at 24 h post-transfection. MiR-139 expression was normalized to U6. *GHR* and *IGF1R* expression was normalized to β-actin. Results are shown as means ± SEM from three independent experiments. Statistical analysis was conducted using one-way ANOVA.“*”, statistical significance was considered at *P*<0.05

To investigate whether expression of *GHR* and *IGF1R* is regulated by miR-139, we measured mRNA levels of miR-139, GHR and IGF1R by qPCR, following over-expression or silencing of miR-139 in cultured BMECs. BMECs were transfected with negative control of miR-139 mimic, miR-139 mimic, negative control of miR-139 inhibitor or miR-139 inhibitor, separately. The Control group was transfected with Lipofectamine 2000. As expected, compared with the Control group, over-expression of miR-139 significantly increased the level of miR-139 (*P* < 0.05) (Fig. 3a), whereas silencing miR-139 decreased it (*P* < 0.05) (Fig. 3b). Then, we examined mRNA levels of *GHR* or *IGF1R* with each treatment. Our qPCR results revealed that over-expression of miR-139 inhibited *GHR* expression by approximately 32% compared with the Control group (*P* < 0.05) (Fig. 3c). However, miR-139 inhibitor treatment increased the level of *GHR* by approximately 37% compared with the Control group (*P* < 0.05) (Fig. 3d). Our qPCR results also showed that over-expression of miR-139 significantly decreased the expression of *IGF1R* by approximately 21% compared with the Control group (*P* < 0.05) (Fig. 3e), whereas loss of miR-139 increased the expression of *IGF1R* by approximately 73% compared with the Control group (*P* < 0.05) (Fig. 3f). Taken together, these results confirmed that miR-139 negatively regulates mRNA levels of *GHR* and *IGF1R*.

### Effects of miR-139 in BMECs

**Fig. 4:**
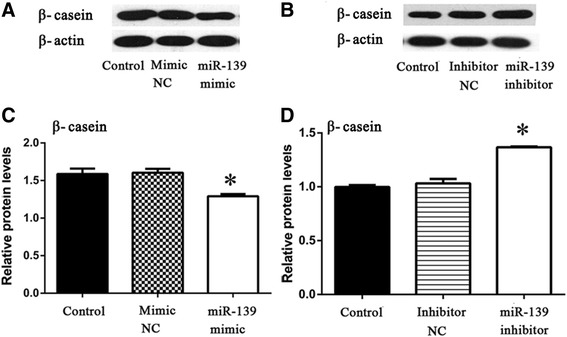
MiR-139 inhibits protein levels of β-casein in BMECs. **a** BMECs were transfected with Lipofectamine 2000 (Control), negative control of miR-139 mimic (Mimic NC) or miR-139 mimic. Western blotting analysis showed the protein levels of β-casein and β-actin at 48 h post-transfection. **b** BMECs were transfected with Lipofectamine 2000 (Control), negative control of miR-139 inhibitor (Inhibitor NC) or miR-139 inhibitor. Western blotting analysis showed the protein levels of β-casein and β-actin at 48 h post-transfection. **c** Relative protein levels were quantified by analyzing scanned blots in panel **a** using Bandscan5.0 software. β-actin was used as a loading control. **d** Relative protein levels were quantified by analyzing scanned blots in panel **b** using Bandscan5.0 software. β-actin was used as a loading control. For **c** and **d**, results are shown as means ± SEM from three independent experiments. Statistical analysis was conducted using one-way ANOVA. “*”, statistical significance was considered at *P*<0.05

The mammary gland in female mammals is responsible for milk production. To determine the role of miR-139 in milk production, we measured the protein levels of β-casein after over-expression or silencing of miR-139 in cultured BMECs. We found that over-expression of miR-139 decreased the protein level of β-casein compared with the Control group (*P* < 0.05) (Fig. 4a and c), whereas silencing miR-139 increased it (*P* < 0.05) (Fig. 4b and d).

**Fig. 5:**
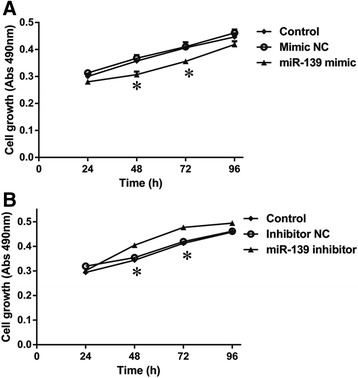
MiR-139 inhibits proliferation of BMECs. **a** BMECs were transfected with Lipofectamine 2000 (Control), negative control of miR-139 mimic (Mimic NC) or miR-139 mimic. Cell proliferation was measured by the MTT assay at 24, 48, 72, or 96 h post-transfection. **b** BMECs were transfected with Lipofectamine 2000 (Control), negative control of miR-139 inhibitor (Inhibitor NC) or miR-139 inhibitor. Cell proliferation was measured by the MTT assay at 24, 48, 72, or 96 h post-transfection. Results are shown as means ± SEM from three independent experiments. Statistical analysis was conducted using one-way ANOVA.“*”, statistical significance was considered at *P*<0.05

Milk production is determined by the number of secretory mammary epithelial cell [25]. Then, we assessed the role of miR-139 on the proliferation of BMECs. The MTT assay revealed that miR-139 significantly decreased the number of BMECs at 48 and 72 h post-transfection with miR-139 mimic in comparison with the Control and Mimic NC groups (*P* < 0.05) (Fig. 5a), whereas silencing miR-139 increased the proliferation of BMECs at 48 and 72 h post-transfection in comparison with the Control and Inhibitor NC groups (*P* < 0.05) (Fig. 5b). These results revealed that miR-139 inhibits cell growth in BMECs.

**Fig. 6:**
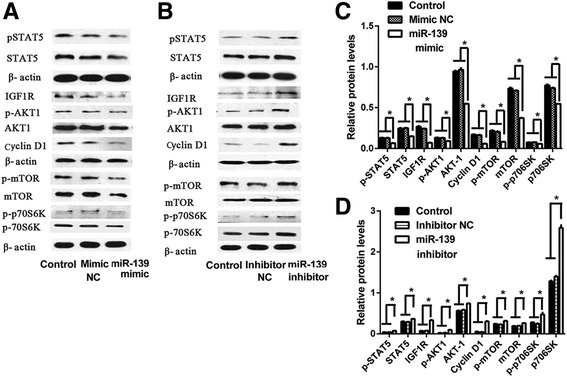
MiR-139 reduces protein levels of major downstream members of GHR and IGF1R signaling in BMECs. **a** BMECs were transfected with Lipofectamine 2000 (Control), negative control of miR-139 mimic (Mimic NC) or miR-139 mimic. Western blotting analysis showed the protein levels of p-STAT5, STAT5, IGF1R, p-AKT1, AKT1, Cyclin D1, p-mTOR, mTOR, p-p70S6K, p70S6K and β-actin at 48 h post-transfection. **b** BMECs were transfected with Lipofectamine 2000 (Control), negative control of miR-139 inhibitor (Inhibitor NC) or miR-139 inhibitor. Western blotting analysis showed the protein levels of p-STAT5, STAT5, IGF1R, p-AKT1, AKT1, Cyclin D1, p-mTOR, mTOR, p-p70S6K, p70S6K and β-actin at 48 h post-transfection. **c** Relative protein levels were quantified by analyzing scanned blots in panel **a** using Bandscan5.0 software. β-actin was used as a loading control. **d** Relative protein levels were quantified by analyzing scanned blots in panel **b** using Bandscan5.0 software. β-actin was used as a loading control. For **c** and **d**, results are shown as means ± SEM from three independent experiments. Statistical analysis was conducted using one-way ANOVA. “*”, statistical significance was considered at *P*<0.05

Next, to explore the underlying molecular mechanism of miR-139, we examined the protein levels of key members of the GHR and IGF1R signaling pathways after over-expression or silencing of miR-139 in cultured BMECs. Western blotting analysis showed that, compared with the Control and Mimic NC groups, over-expression of miR-139 reduced protein levels of p-STAT5 and STAT5, which are key members in the GHR signaling pathway (*P* < 0.05) (Fig. 6a and c); IGF1R, p-AKT1, AKT1 and Cyclin D1, which are key members in the IGF1R signaling pathway (*P* < 0.05) (Fig. 6a and c); and p-mTOR, mTOR, p-p70S6K and p70S6K, which are key members in both GHR and IGF1R signaling pathways (*P* < 0.05) (Fig. 6a and c). Moreover, compared with the Control and Ihibitor NC groups, silencng miR-139 increased the levels of these proteins (*P* < 0.05) (Fig. 6b and d). These results demonstrated that miR-139 down-regulates β-casein synthesis and mammary epithelial cells proliferation by influencing the GHR and IGFIR signaling pathways in BMECs.

### MiR-139 also regulates β-casein synthesis and IGF1R signaling by influencing *GHR*

**Fig. 7:**
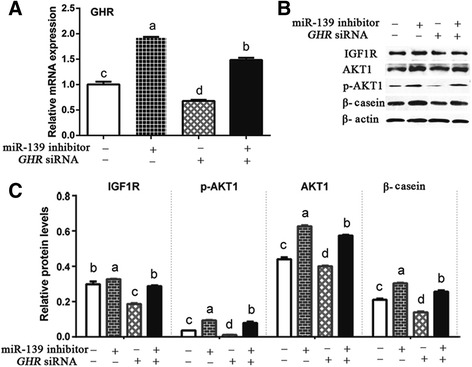
MiR-139 also regulates synthesis of β-casein and IGF1R signaling by influencing *GHR*. BMECs were transfected with (without) *GHR* siRNA and (or) miR-139 inhibitor. **a** GHR mRNA levels were examined by qPCR at 24 h post-transfection in cultured BMECs. GHR expression was normalized to β-actin. **b** Western blotting analysis showed the levels of IGF1R, p-AKT1, AKT1, β-casein, and β-actin at 48 h post-transfection in cultured BMECs. **c** Relative protein levels were quantified by analyzing scanned blots in panel **b** using Bandscan5.0 software. β-actin was used as a loading control. For **a** and **c**, results are shown as means ± SEM from three independent experiments. Statistical analysis was conducted using one-way ANOVA. Bars with different lowercase letters are significantly different (*P* < 0.05)

We next sought to determine the relationship among miR-139, GHR, and IGF1R. As we have shown, silencing miR-139 significantly increased mRNA levels of *GHR* (*P* < 0.05) (Fig. 7a), and protein levels of IGF1R, p-AKT1, AKT1, and β-casein in comparison with the Control group (*P* < 0.05) (Fig. 7b and c). While, silencing *GHR* by siRNA significantly decreased the mRNA levels of *GHR* in comparison with the Control group (*P* < 0.05) (Fig. 7a). Moreover, the loss of *GHR* expression also contributed to decreased protein levels of IGF1R, p-AKT1, AKT1, and β-casein in comparison with the Control group (*P* < 0.05) (Fig. 7b and c). In addition, when *GHR* and miR-139 were both silenced, mRNA level of *GHR* and protein levels of IGF1R, p-AKT1, AKT1, and β-casein increased relative to those of the group with silenced *GHR* (*P* < 0.05) (Fig. 7a–c). These results indicated that IGF1R signaling is also regulated by *GHR*. However, the expression and effects of *GHR* can be influenced by miR-139.

## Discussion

The mammary gland gains the function of milk production during lactation [26]. Our previous results from microRNA microarrays showed that the expression of some miRNAs, such as miR-139, is down-regulated on transition from pregnancy to lactation in mammary tissues from mouse [11] and dairy cow (data unpublished). Since miRNAs can trigger inhibition of protein translation [4, 27, 28], down-regulation of certain miRNAs during lactation indicates that they may play important roles related to lactation [29]. We observed that the expression of miR-139 is reduced in dairy cow mammary gland from pregnancy to lactation. Therefore, we hypothesized that miR-139 may be involved in lactation of dairy cows and tested this hypothesis. Out of potential target genes for miR-139, we prioritized *GHR* and *IGF1R* for further studies based on their roles in lactation processes. Our dual luciferase reporter assay indicated that miR-139 binds to *GHR* and *IGF1R* by their 3**′**-UTRs. Furthermore, we observed that mRNA levels of GHR and IGF1R were inversely correlated with miR-139 expression in cultured BMECs. These results confirmed that *GHR* and *IGF1R* are target genes of miR-139 in dairy cow mammary gland.

GHR activates signaling pathways including the Janus kinase2/signal transducer and activator of transcription (JAK2/STAT), mitogen activated protein kinase (MAPK), and the phosphatidylinositol-3-kinase (PI3K) pathways [14]. In particular, the JAK-STAT5 pathway is the main pathway through which GH influences the expression of genes encoding milk proteins such as β-casein, an important differentiation marker in mammary epithelial cells [16, 30, 31]. Moreover, GH influences protein metabolism through changes in the mTOR signaling pathway in bovine mammary gland [9]. It is worth noting that the mTOR signaling pathway can also be influenced by IGF1R. There are two classical signaling pathways regulated by IGF1R, the PI3K-AKT and RAS/RAF/MAPK pathways [32]. IGF1R regulates protein synthesis through the PI3K-AKT1-mTOR pathway [33]. After phosphorylation of mTOR, the mTOR pathway regulates the translation process by directly phosphorylating the translational regulators eukaryotic translationinitiation factor 4E (eIF4E) binding protein 1 (4E–BP1) and S6 kinase 1 (S6 K1), which in turn promote protein synthesis [19, 34]. In our study, we revealed that miR-139 reduced protein levels of β-casein. In addition, we found that miR-139 inhibited not only the activity of downstream signaling molecules of the GHR and IGF1R pathways, but also the total protein levels of key molecules of these pathways. This phenomenon has also been found by others [24, 35]. These observations suggest that miR-139 likely inhibits synthesis of β-casein through GHR and IGF1R signaling in BMECs.

In general, the number of secretory mammary epithelial cells determines milk production in a mammary gland, and the balance between proliferation and apoptosis controls the number of secretory mammary epithelial cells. Both proliferation and apoptosis produce effects gradually throughout the lactation period [25]. Previous studies showed that IGF1R can stimulate cell proliferation and inhibit apoptosis through the PI3K/AKT and MAPK pathways [36]. Our study revealed that miR-139 down-regulated expression of IGF1R and PI3K/AKT members. Moreover, in our cell proliferation assay, miR-139 significantly inhibited the growth of BMECs. Cyclin D is a known cell cycle regulator which indirectly stimulates the expression of various cell cycle promoters [37]. Previous studies showed that IGF1R induces elevated protein levels of Cyclin D1 through binding of the lymphoid Enhancer-Binding Factor 1 (LEF1) transcription factor in the nucleus [38]. Then, we determined the expression of Cyclin D1 by western blotting analysis. Our results showed that miR-139 decreased the protein level of Cyclin D1 in cultured BMECs. These results suggest that miR-139 also reduces the number of BMECs via IGF1R signaling.

In this study, we determined that *GHR* and *IGF1R* are target genes of miR-139 and that miR-139 inhibits synthesis of β-casein through down-regulating both GHR and IGF1R signaling. However, there is another connection between GHR and IGF1R. As we know, the binding of GHR to its ligand regulates IGF1 production by activating the STAT5b signaling cascade [39, 40]. Moreover, postnatal IGF1 production is predominantly regulated by GH [41]. These data indicate that the effect of GHR on the mammary gland may be exerted via IGF1R. Then, we investigated the relationship among miR-139, GHR, and IGF1R. As a result, we revealed that GHR is a regulator of IGF1R signaling because silencing *GHR* decreased protein levels of β-casein, IGF1R, and key members of the IGF1R pathway. Additionally, we found that co-silencing miR-139 and *GHR* rescued the expression of *GHR* and *GHR* silencing effects. These data suggest that miR-139 also regulates synthesis of β-casein and IGF1R signaling by influencing GHR.

## Conclusions

In conclusion, we have revealed that miR-139 is down-regulated in lactating dairy cow mammary glands and that miR-139 inhibits β-casein synthesis and proliferation by targeting the GHR and IGF1R signaling pathways in BMECs. This finding suggests that miR-139 may act as a potent inhibitor in lactation.

## Abbreviations

AKT1: Protein kinase B
BMEC: Bovine mammary epithelial cell
DIM: Day in milk
DMEM: Dulbecco’s modified Eagle’s medium
DMSO: Dimethyl sulfoxide
eIF4E: Eukaryotic translationinitiation factor 4E
FBS: Fetal bovine serum
GH: Growth hormone
GHR: Growth hormone receptor
IGF1: Type I insulin-like growth factor
IGF1R: Type I insulin-like growth factor receptor
JAK: Janus kinase
LEF1: Lymphoid Enhancer-Binding Factor 1
MAPK: Mitogen activated protein kinase
mTOR: Mammalian target of rapamycin.
MTT: 3-(4,5-Dimethylthiazol-2-yl)-2,5- diphenyltetrazolium bromide.
Mut: Mutant.
p70S6K: p70-S6 Kinase.
PI3K: Phosphatidylinositol-3-kinase.
qPCR: Quantitative Real-time Polymerase Chain Reaction.
SDS-PAGE: Sodium dodecyl sulfate polyacrylamide gel electrophoresis.
SEM: Standard error of mean.
STAT5: Signal transducer and activator of transcription-5.
UTR: Untranslated region.
WT: Wild-type.

## References

[1] Yonemori M, Seki N, Yoshino H, Matsushita R, Miyamoto K, Nakagawa M, Enokida H (2016). Dual tumor-suppressors miR-139-5p and miR-139-3p targeting matrix metalloprotease 11 in bladder cancer. Cancer Sci.

[2] Qiu G, Lin Y, Zhang H, Wu D (2015). miR-139-5p inhibits epitheliale mesenchymal transition, migration and invasion of hepatocellular carcinoma cells by targeting ZEB1 and ZEB2. Biochem Biophys Res Commun.

[3] Chen X, Gao C, Li H, Huang L, Sun Q, Dong Y, Tian C, Gao S, Dong H, Guan D, Hu X, Zhao S, Li L, Zhu L, Yan Q, Zhang J, Zen K, Zhang CY (2010). Identification and characterization of microRNAs in raw milk during different periods of lactation, commercial fluid, and powdered milk products. Cell Res.

[4] Hasseine LK, Hinault C, Lebrun P, Gautier N, Paul-Bellon R, Van Obberghen E (2009). miR-139 impacts FoxO1 action by decreasing FoxO1 protein in mouse hepatocytes. Biochem Biophys Res Commun.

[5] Zhang HD, Sun DW, Mao L, Zhang J, Jiang LH, Li J, WuY JH, Chen W, Wang J, Ma R, Cao HX, Wu JZ, Tang JH (2015). MiR-139-5p inhibits the biological function of breast cancer cells by targeting Notch1 and mediates chemosensitivity to docetaxel. Biochem Biophys Res Commun.

[6] Liu R, Yang M, Meng Y, Liao J, Sheng J, Pu Y, Yin L, Kim SJ. Tumor-suppressive function of miR-139-5p in esophageal squamous cell carcinoma. PLoS One. 2013; 10.1371/journal.pone.0077068. 10.1371/journal.pone.0077068 PMC3799985 24204738

[7] Zhang L, Dong Y, Zhu N, Tsoi H, Zhao Z, Wu CW, Wang K, Zheng S, Ng SS, Chan FK, Sung JJ, Yu J. microRNA-139-5p exerts tumor suppressor function by targeting NOTCH1 in colorectal cancer. Mol Cancer. 2014; doi:10.1186/1476-4598-13-124. 10.1186/1476-4598-13-124 PMC4065091 24885920

[8] Xu W, Hang M, Yuan CY, FL W, Chen SB, Xue K (2015). MicroRNA-139-5p inhibits cell proliferation and invasion by targeting insulin-like growth factor 1 receptor in human non-small cell lung cancer. Int J Clin Exp Pathol.

[9] Wong CC, Wong CM, Tung EK, SL A, Lee JM, Poon RT, Man K, Ng IO (2011). The microRNA miR-139 suppresses metastasis and progression of hepatocellular carcinoma by down-regulating rho-kinase 2. Gastroenterology.

[10] Krishnan K, Steptoe AL, Martin HC, Pattabiraman DR, Nones K, Waddell N, Mariasegaram M, Simpson PT, Lakhani SR, Vlassov A, Grimmond SM, Cloonan N (2013). miR-139-5p is a regulator of metastatic pathways in breast cancer. RNA.

[11] Wang C, Li Q (2007). Identification of differentially expressed microRNAs during the evelopment of Chinese murine mammary gland. J Genet Genomics.

[12] Hayashi AA, Nones K, Roy NC, McNabb WC, Mackenzie DS, Pacheco D, McCoard S (2009). Initiation and elongation steps of mRNA translation are involved in the increase in milk protein yield caused by growth hormone administration during lactation. J Dairy Sci.

[13] Lombardi S, Honeth G, Ginestier C, Shinomiya I, Marlow R, Buchupalli B, Gazinska P, Brown J, Catchpole S, Liu S, Barkan A, Wicha M, Purushotham A, Burchell J, Pinder S, Dontu G (2014). Growth hormone is secreted by normal breast epithelium upon progesterone stimulationand increases proliferation of stem/progenitor cells. Stem Cell Reports.

[14] Perry JK, Mohankumar KM, Emerald BS, Mertani HC, Lobie PE (2008). The contribution of growth hormone to mammary neoplasia. J Mammary Gland Biol Neoplasia.

[15] Sakamoto K, Komatsu T, Kobayashi T, Rose MT, Aso H, Hagino A, Obara Y (2005). Growth hormone acts on the synthesis and secretion of alpha-casein in bovine mammary epithelial cells. J Dairy Res.

[16] Postel-Vinay MC, Kelly PA (1996). Growth hormone receptor signalling. Bailliere Clin Endocrinol Metab.

[17] Ilkbahar YN, Thordarson G, Camarillo IG, Talamantes F (1999). Differential expression of the growth hormone receptor and growth hormone-binding protein in epithelia and stroma of the mouse mammary gland at various physiological stages. J Endocrinol.

[18] Pal S, Woodford K, Kukuljan S, Ho S (2015). Milk intolerance, Beta-casein and lactose. Nutrients.

[19] Sciascia Q, Pacheco D, McCoard SA (2013). Increased milk protein synthesis in response to exogenous growth hormone is associated with changes in mechanistic (mammalian) target of rapamycin (mTOR)C1-dependent and independent cell signaling. J Dairy Sci.

[20] Divisova J, Kuiatse I, Lazard Z, Weiss H, Vreeland F, Hadsell DL, Schiff R, Osborne CK, Lee AV (2006). The growth hormone receptor antagonist pegvisomant blocks both mammary gland development and MCF-7 breast cancer xenograft growth. Breast. Cancer Res Treat.

[21] Felice DL, El-Shennawy L, Zhao S, Lantvit DL, Shen Q, Unterman TG, Swanson SM, Frasor J (2013). Growth hormone potentiates 17β-estradiol-dependent breast cancer cell proliferation independently of IGF-I receptor signaling. Endocrinology.

[22] Fürstenberger G, Morant R, Senn HJ (2003). Insulin-like growth factors and breast cancer. Onkologie.

[23] Cui Y, Liu Z, Sun X, Hou X, Qu B, Zhao F, Gao X, Sun Z, Li Q (2015). Thyroid hormone responsive protein spot 14 enhances lipogenesis in bovine mammary epithelial cells. In Vitro Cell Dev Biol Anim.

[24] Zhao F, Liu C, Hao YM, Qu B, Cui YJ, Zhang N, Gao XJ, Li QZ (2015). Up-regulation of integrin α6β4 expression by mitogens involved in dairy cow mammary development. In Vitro Cell Dev Biol Anim..

[25] Murney R, Stelwagen K, Wheeler TT, Margerison JK, Singh K (2015). The effects of milking frequency in early lactation on milk yield, mammary cell turnover, and secretory activity in grazing dairy cows. J Dairy Sci.

[26] Knight CH, Peaker M (1982). Development of the mammary gland. J ReprodFertil.

[27] Zhang HD, Jiang LH, Sun DW, Li J, Tang JH (2015). MiR-139-5p:promising biomarker for cancer. Tumour Biol.

[28] Ruelas DS, Chan JK, Oh E, Heidersbach AJ, Hebbeler AM, Chavez L, Verdin E, Rape M, Greene WC (2015). MicroRNA-155 reinforces HIV latency. J Biol Chem.

[29] Zhou Y, Gong W, Xiao J, Wu J, Pan L, Li X, Wang X, Wang W, Hu S, Transcriptomic YJ (2014). Analysis reveals key regulators of mammogenesis and the pregnancy-lactationcycle. Sci China Life Sci.

[30] Chiba T, Maeda T, Sanbe A, Kudo K (2016). Serotonin suppresses β-casein expression via PTP1B activation in human mammary epithelial cells. Biochem Biophys Res Commun.

[31] Postel-Vinay MC, Finidori J (1995). Growth hormone receptor: structure and signal transduction. Eur J Endocrinol.

[32] Chitnis MM, Yuen JS, Protheroe AS, Pollak M, Macaulay VM (2008). The type 1 insulin-like growth factor receptor pathway. Clin Cancer Res.

[33] Iams WT, Lovly CM (2015). Molecular pathways: clinical applications and future direction of insulin-like growth Factor-1 receptor pathway blockade. Clin Cancer Res.

[34] Laplante M, Sabatini DM (2012). mTOR signaling in growth control and disease. Cell.

[35] Ao J, Wei C, Si Y, Luo C, Lv W, Lin Y, Cui Y, Gao X (2015). Tudor-SN Regulates milk synthesis and proliferation of bovine mammary epithelial cells. Int J Mol Sci.

[36] de Groot S, Charehbili A, van Laarhoven HW, Mooyaart AL, Dekker-Ensink NG, van de Ven S, Janssen LG, Swen JJ, Smit VT, Heijns JB, Kessels LW, van der Straaten T, Böhringer S, Gelderblom H, van der Hoeven JJ, Guchelaar HJ, Pijl H, Kroep JR, Dutch Breast Cancer Research Group (2016). Insulin-like growth factor 1 receptor expression and IGF1R 3129G > T polymorphism are associated with response to neoadjuvant chemotherapy in breast cancer patients: results from the NEOZOTAC trial (BOOG 2010–01). Breast Cancer Res.

[37] Park JH, Darvin P, Lim EJ, Joung YH, Hong DY, Park EU, Park SH, Choi SK, Moon ES, Cho BW, Park KD, Lee HK, Kim MJ, Park DS, Chung IM, Yang YM. Hwanggeumchal sorghum induces cell cycle arrest, and suppresses tumor growth and metastasis through Jak2/STAT pathways in breast cancer xenografts. PLoS One. 2012; doi:10.1371/journal.pone.0040531. 10.1371/journal.pone.0040531 PMC3391253 22792362

[38] Warsito D, Sjöström S, Andersson S, Larsson O, Sehat B (2012). Nuclear IGF1R is a transcriptional co-activator of LEF1/TCF. EMBO Rep.

[39] Hwa V, Nadeau K, Wit JM, Rosenfeld RG (2011). STAT5b deficiency: lessons from STAT5b gene mutations. Best Pract Res Clin Endocrinol Metab.

[40] Feigerlova E, Hwa V, Derr MA, Rosenfeld RG (2013). Current issues on molecular diagnosis of GH signaling defects. Endocr Dev.

[41] Hwa V, Fang P, Derr MA, Fiegerlova E, Rosenfeld RG (2013). IGF-I in human growth: lessons from defects in the GH-IGF-I axis. Nestle. NutrInst workshop. Ser.

